# Fostering the conduct of ethical and equitable research practices: the imperative for integrated knowledge translation in research conducted by and with indigenous community members

**DOI:** 10.1186/s40900-018-0131-1

**Published:** 2018-11-26

**Authors:** Janet Jull, Melody Morton-Ninomiya, Irene Compton, Annie Picard

**Affiliations:** 1 School of Rehabilitation Therapy, Queen’s University, 31 George Street, Kingston, ON K7L 3N6 Canada; 2 Ottawa Hospital Research Institute & University of Ottawa, 501 Smyth Road, Ottawa, ON Canada; 3 Centre for Addiction and Mental Health, Well-Living House, St. Michael’s Hospital, 30 Bond Street, Toronto, ON Canada; 4 Minwaashin Lodge – Aboriginal Women’s Support Centre, 1155 Lola Street, Ottawa, ON Canada; 5 Innu Round Table, 7 Peenamin Drive, Sheshatshiu, Newfoundland and Labrador, Canada

**Keywords:** Integrated knowledge translation, Indigenous knowledge translation, Co-creation, Collaboration, Ethics, Equity

## Abstract

**Plain English summary:**

Integrated knowledge translation is a research approach in which researchers work as partners with the people for whom the research is meant to be of use. A partnered approach can support the use of Indigenous ways of knowing in health research that may then be used in health care. This is important as current health care models do not often support Indigenous values, ways of knowing, and care practices. We describe 1) why it is necessary to co-create knowledge that includes the voices of Indigenous community members, 2) how integrated knowledge translation is a way of doing research that includes many views and 3) how integrated knowledge translation can help those involved in research to agree upon and uphold ethical ways of doing research. Integrated knowledge translation may be used to include Indigenous ways of knowing into mainstream health research and to improve health systems. The use of an integrated knowledge translation approach in research may guide researchers to be research partners with Indigenous people and groups. Integrated knowledge translation may be a way to do research that is respectful and to ensure that Indigenous ways of knowing are included in both health research and health care systems.

**Abstract:**

**Background**

Indigenous people are affected by major health issues at much higher rates than for general populations, and Western health care models do not respond or align with Indigenous values, knowledge systems, and care practices. Knowledge translation (KT) describes ways of moving knowledge from theory into health systems’ applications, although there are limitations and concerns related to the effectiveness and contributions of Western-informed approaches to research and KT practices that promote health with Indigenous groups. Integrated KT is an approach to research that engages researchers with the people for whom the research is ultimately meant to be of use (“knowledge users”) throughout the entire research process. Integrated KT is done in ways that knowledge users may define as useful, relevant, and applicable in practice, and may also be viewed as complementary to Indigenous health research principles.

**Main**

In this paper, we raise and discuss questions posed to researchers by Indigenous knowledge-users about perspectives on health research, researchers, and research institutions, and focus on the role and ethical imperative for integrated KT in Indigenous health research. We describe: 1) why it is necessary to co-create knowledge that includes the voices of Indigenous community members within institutional academic spaces such as universities; 2) how integrated KT accommodates Indigenous and Western-informed perspectives in community-research partnerships throughout the research process; and 3) how an integrated KT approach can help those involved in research to define, agree upon and uphold ethical practices. We argue that integrated KT as a collaborative research practice can create opportunities and space within institutional academic settings for different knowledges to coexist and improve health systems. Most importantly, we argue that integrated KT in Indigenous research contexts includes Indigenous KT.

**Conclusion**

The use of integrated KT facilitates opportunities to further define and develop understandings about collaborative approaches to research with Indigenous research partners and that may contribute to respectful inclusion of Indigenous KT practices and processes within institutional academic settings. In the pursuit of useful, relevant and applicable knowledge, those within Western research and health systems must examine and expand upon collaborative approaches to KT.

## Background

There are unfair, avoidable, and well-documented differences (“inequities”) in health status between Indigenous and non-Indigenous populations in Canada, Australia, New Zealand, and the United States: these health inequities are the result of a combination factors, including general socioeconomic and social, historical and political factors and that are particular to the affected groups [1]. Colonialism and self-determination are key determinants of Indigenous peoples’ health [2]. Historical events, policies, and attitudes regarding Indigenous people have been used to control, assimilate, and impose a lifestyle that is based on European Settler ideologies. Indigenous peoples have experienced forced relocation and settlement, been subjected to the removal of children from families to attend religious institutions and government-run residential schools, and had their autonomy subverted to develop dependencies upon government-regulated supports and services – in health, education, and child welfare among others [3]. Many colonial policies and practices endure today; some are overt while others are insidious. The result is that many Indigenous people experience the effects of colonialism and racism on a daily basis [4, 5] and this is reflected in health outcomes.

Indigenous people are affected by major health issues at much higher rates than non-Indigenous people [1]. For example, Indigenous populations in Canada have lower life expectancies at birth than those in the general populations [6, 7] and the differences in socio-economic status are associated with health gaps between Indigenous and general populations [8, 9]. In addition to experiencing racism, Indigenous people experiencing health challenges frequently only have access to Western health care services [5]. Western health care models rarely reflect values, knowledge systems, and care practices that align with local Indigenous cultures. Indigenous people who use health services [1, 2] often encounter barriers within mainstream health systems [3, 4, 8, 9] and consequently, the uptake of health services can be poor. There is a need to build evidence that can be used in health systems to promote processes and practices that are both culturally safe and appropriate and defined as useful, relevant and applicable by those whom the research is meant to benefit, as well as by those who deliver health services.

Knowledge translation (KT) is a term frequently used to describe ways of moving knowledge from theory into application. In Canada, terms used to refer to this general concept of KT in other jurisdictions also call it knowledge exchange and transfer or knowledge mobilization [10]. The term ‘knowledge translation’ is the mandate of the federal funding body, the Canadian Institute of Health Research [11]: “a dynamic and iterative process that includes synthesis, dissemination, exchange and the ethically sound application of knowledge to improve the health of Canadians, provide more effective health services and products and to strengthen the health care system” (para 4). How knowledge is produced by researchers has been suggested as creating or contributing to what is referred to as the know-do gap [12, 13], referring to the failure of research to be acted upon. The ultimate aim of KT is to bridge the know-do gap that exists between knowledge development and uptake, and is an approach to implement an interactive process of knowledge exchange between health researchers and people directly affected by research and/or those who can act on the findings such as community members, healthcare providers, et cetera (“knowledge users”) [14]. *Integrated knowledge translation* (“integrated KT”) has been proposed as an approach to address the problematic issues with the process of knowledge generation inherent in traditional research methods and knowledge production.

Integrated KT is an approach to research within Western-informed knowledge systems that engages knowledge users with researchers throughout the entire research process - from defining the research question to applying the findings [15]. With integrated KT, both the knowledge user and researcher partner(s) are acknowledged as bringing valuable expertise to the research process. For example, the knowledge user may provide contextual information and the researcher may contribute information on research methodologies and methods. As a research approach, integrated KT can be used with a range of theoretical research traditions and (ideally) emphasizes researcher-knowledge user collaboration in every step of the research process: the determination of research questions, decisions about the methodology and methods, data collection and analysis, and interpretation and participation in the dissemination of the research findings [16]. Research that is undertaken in collaboration with knowledge users that uses an integrated KT approach fosters democratic processes of knowledge production [17]. Integrated KT has the potential to produce knowledge that can be put into practice, as it is done with the expectation and aim for the research outputs to be relevant, useful, and able to be applied in practice. Expecting knowledge can be put into practices makes it more likely that research will be applied in practice and policy [18].

In this paper, we raise and discuss questions from Indigenous knowledge-users’ perspectives on health research, researchers, and research institutions. We focus on the role and ethical imperative for integrated KT in Indigenous health research, with attention to the relevance in Western-informed health systems. We describe: 1) why it is necessary to co-create knowledge that is inclusive of Indigenous community members within institutional academic spaces such as universities; 2) how integrated KT accommodates Indigenous and Western-informed perspectives in community-research partnerships throughout the research process; and 3) how to define and uphold ethical practices that are agreed upon by those involved in an integrated KT program of research. We argue that integrated KT as a collaborative research practice can create opportunities and space within institutional academic settings for different knowledges to coexist and improve health systems. Most importantly, we argue that integrated KT in Indigenous research contexts includes Indigenous KT.

## Main text

We are community-researcher pairs (community member and researcher partners) with experience working from within an integrated KT approach (IC, JJ and AP, MMN). IC is a First Nations member, Elder, and one of the founders of Minwaashin Lodge in Ottawa Ontario Canada, an urban Indigenous community. Minwaashin Lodge provides intervention services and programs to First Nations, Inuit and Métis women, children and youth who are survivors of family violence and/or the residential school system, including the intergenerational impacts of violence against Indigenous people in Canada. JJ is a researcher of Euro-Canadian descent and who, as a health care provider, was concerned by the ways in which health systems failed to meet the needs of the people it was meant to benefit. In full collaboration with Minwaashin Lodge leaders, JJ collaborated in a series of research studies aimed at enhancing opportunities for participation of First Nations, Inuit and Métis women in their health decisions. AP is an Innu health leader who lives in a rural and remote Innu First Nation community of 1200 people in Labrador on Canada’s east coast. AP was primarily raised by her grandmother who taught her a lot about the Innu way of life, living off the land. Her rich experience of being raised with strong Innu values, language, and knowledge has motivated her to do paid work in her home community. MMN is a researcher of Japanese and Euro-Canadian descent and a parent to an Innu child who was placed ‘in care’ due to medical needs that could not be met in her birth family’s remote community home. As a result, MMN has extended family connections through her Innu daughter’s birth family to many people living in a different Innu First Nation community from AP, in Labrador. MMN and AP started working together when MMN was conducting doctoral research on fetal alcohol spectrum disorder (FASD) with Innu First Nation’s stakeholder guidance. Since then, AP and MMN have worked together on multiple community-initiated projects and priorities. In the case of both community research pairs, each was working to address health concerns identified as a priority by the community.

This paper reflects the authors’ ongoing efforts, adding to the literature on collaborative research practices. Our paper is situated within community-researcher pair discussions comprised of shared questions, accounts and reflections about their respective experiences with health research.

### The role and ethical imperative for integrated KT in indigenous health research: Attending to the relevance in Western-informed health systems

There is potential for integrated KT, as a collaborative research practice, to create opportunities for research partnerships between Indigenous community members and researchers; provide a space for different knowledges to coexist; and, enable the application of this knowledge in health systems. An integrated KT approach is intended to promote the conduct of research wherein research partners can view their knowledge systems as respected and included. Such respectful and inclusive research conduct does not happen automatically. In Canada, researchers who wish to engage in research partnerships with Indigenous peoples are expected to adhere to principles such as the ‘four R’s of research which include respect, reciprocity, relevance, and responsibility [19] plus a subsequent “fifth R” on relationships [20]; and the Ownership, Control, Access, and Possessions principles^1^ [21]. These principles require careful application [22–24] and align with the integrated KT approach. Principles for research with Indigenous people and integrated KT both place an emphasis on reciprocity and knowledge sharing [25] that result in collaborative knowledge user-researcher relationships, disrupting potential divisions between those who do research and those who are participants in the research [17]. The aim of an integrated KT approach is to strive to conduct research in ways that knowledge users themselves define as ethical and acceptable [26, 27]. As a collaborative research approach, integrated KT holds promise for enhancing community research partnerships that are compatible with both Indigenous and Western-informed knowledge systems. As a research approach, rather than a checklist of criteria, integrated KT promotes principled research processes throughout the conduct of the research.

How people follow guidelines for ethical research involving Indigenous people and adhere to Indigenous health research principles may vary depending on the people involved, the nature of the study, the intent of the study, the history and relationship between researchers and community, and many other factors [22, 28]. Researchers are often forced to rely on documents such as the CIHR Aboriginal Peoples’ Health document “Aboriginal Knowledge Translation: Understanding and Respecting the Distinct Needs of Aboriginal Communities in Research” [29] that highlight the importance and ethical responsibilities of Indigenous KT. The Indigenous knowledge translation document is literature that draws attention to the differences between knowledge systems and their assumptions of Indigenous and non-Indigenous KT models and practices [29, 30]. Universities, for example, are identified as places that “tend to acculturate and conscript different kinds of knowledge into their own existing categories for what can be known, how knowledge can be organized, and what forms of knowledge are legitimate and credible” (p. 142) [31]. Fortunately, the progression of mainstream KT includes different approaches, such as integrated KT, that is both necessary and the precursor to creating opportunities for many ways of knowing, such as Indigenous KT. As integrated KT is conducted from within partnerships, it is deliberately inclusive of many ways of knowing and that can lead to co-creation of knowledge that is useful, relevant, and applicable within Western healthcare systems. The distinction between integrated and Indigenous KT are discussed later in this paper.

### Why it is necessary to co-create knowledge that is inclusive of indigenous community members within institutional academic spaces

Integrated KT aims to foster the co-creation of knowledge that is the result of researcher and knowledge user expertise. Implicit in the integrated KT process is collaboration between researchers and knowledge users, whereby each participant brings valuable insights and knowledge. It is important to co-create knowledge that is inclusive of Indigenous viewpoints, as within mainstream academic settings, Western-informed forms of knowledge are privileged above “other” forms of knowledge. As well, current within mainstream academic and healthcare systems, the application of Western-derived knowledge alone is not reflected in the health and well-being of Indigenous peoples [1]. It is critical that Indigenous groups identify and develop knowledge that is useful, relevant, and applicable in Indigenous people’s contexts. To do this, researchers must find ways to co-create knowledge with Indigenous partners, and to begin, there is a need for clarity about the research endeavour. That is, potential Indigenous partners in research need to know the ways in which knowledge will be shared and used. We suggest researchers must be forthcoming and if they are not, knowledge users must ask researchers the following questions:

- Where is the knowledge being shared?
- What are your [researcher] intentions?
- How will sharing our knowledge help our community or other communities?

Ermine et al. [32] writes about the importance of creating an *ethical space* in which disparate cultural practices and ways of knowing and being can bridge the divide between researcher and knowledge user by being transparent – with open discussion – about research intentions, values, and underlying and explicit assumptions. Creating ethical spaces can allow for multiple ways of knowing and doing – to coexist [33]. For example, JJ was supported by knowledge user research partners to present the results of interviews conducted with community members in the form of a Medicine Wheel, defined by the leaders at Minwaashin Lodge as an appropriate way of sharing knowledge. JJ was taught about the Medicine Wheel by the knowledge user research partners about how to present the information in a respectful way that would be meaningful and accessible to other Indigenous people and conveying teachings to non-Indigenous people [34].

As another example, at the end of the FASD study, community leaders wanted to create a community asset map of all health-related supports and services provided to the community. A visually engaging community asset map called the “Health and Healing Map” was co-created through an iterative process [35–37]. AP was involved in the conceptual grouping of information and MMN created the Health and Healing Map. Community leaders that were consulted during the development of the map later asked that the map be shared and vetted at a health forum that brought together members from both Innu communities in Labrador. In the end, Health and Healing Maps were developed for both Innu communities and were shared with every household so that all community members were aware of the range of services available to them, including contact information and physical locations.

Co-creation and sharing of knowledge must be undertaken in such a way that all parties who are involved in the partnership can identify the process as inclusive and equitable. Given the disparate level of equity between Western society and Indigenous peoples, the onus is on researchers and their knowledge user partners, research institutions and research funders to: support and operate within collaborative partnerships with community collaborators; ensure that time and resources are available to develop and sustain relationships; and, openly discuss and agree upon how knowledge will be co-created and shared. For this reason,there is a need for an integrated KT approach.

### How integrated KT accommodates indigenous and Western-informed perspectives in community-research partnerships: True versus token collaboration

There is a growing interest in collaborative approaches to generating knowledge between knowledge users and researchers and that lead to “co-created” knowledge [38], which is more likely to meet the needs of and be acceptable to health systems’ knowledge users. Collaborative research has been found to create important opportunities for real change, frequently involve knowledge users, increase influence on behaviours of knowledge user partners, and facilitate knowledge application in real-world settings [39]. Authors IC and AP have asked the following questions to JJ and MMN in the process of refining the content of this paper (Table 1):

- Why don’t researchers collaborate in research studies more often?
- Whose research is it?
- Why aren’t researchers taking time to understand how the present reality is connected to our history?
- Why aren’t researchers interested in having long-term relationships with us?

**Table 1:** True versus token collaboration

Questions Aimed at Researchers	Observations and Lessons Learned
Why don’t researchers collaborate on research studies more often?	• Incorporating capacity building helps to facilitate intergenerational success. • Research partnerships with community can offer capacity building opportunities for community members and researchers alike. • Leadership from community is needed to establish standards for co-facilitated studies.
Whose research is it?	• Indigenous community and researchers must discuss and be transparent about who benefits and how research process and findings will be shared. • How Indigenous people and communities are portrayed is very important. Elders and other Indigenous counsellors must be involved to ensure that representations and descriptions of people and culture are appropriate. • Research should be strengths-based. A problem-based approach with a focus on negatives and challenges, is not helpful.
Why aren’t researchers taking time to understand how the present reality is connected to our history?	• Researchers must take time to learn the history of Indigenous people/communities. • Knowing history can put current realities into context. • Researchers need to participate in community events and invite community members to be part of research events, to honour one another’s ways.
Why aren’t researchers interested in having a long- term relationships with us?	• Indigenous people trust researchers that demonstrate that they genuinely care about the (Indigenous) community, sustained over time. • Researchers that genuinely care are interested in getting to know the specific Indigenous community, what the community has been through, and know how to be respectful.

In our work, we have found that genuine involvement between researchers and knowledge user partners has fostered research that both researcher and Indigenous knowledge user partners define as successful. The process of building and enacting collaborative research demonstrates respect and values knowledge held and contributed by knowledge users and authentic research collaboration is a critical feature of this approach. There are increasing opportunities to leverage knowledge and experience on both sides of the researcher/knowledge user relationship to inform research and that include KT approaches. For example, many contemporary researchers have taken a strong interest in Innu culture, knowledge, traditions, language, and legends. Complimentarily, there are well-respected Innu community members who are rich with Innu knowledge, much of which would be analogous with Western expertise in archeology, anthropology, geology, history, language, and genealogy. Researchers often come into Indigenous communities to befriend Innu knowledge holders, going on to publish and appropriate ownership of Indigenous knowledge with often little more than perfunctory acknowledgement of their Indigenous sources. Ironically, many scholars and researchers use such appropriated knowledge in academia to establish their credentials as ‘experts’ on Innu society or culture, instead of the community knowledge holders who have shared their expertise and who are the sources of the academic knowledge. Few Indigenous knowledge holders are funded or invited to co-present at conferences or in lectures. Indigenous leaders have described this history of knowledge appropriation as particularly hurtful and exploitive [40, 41]. The absence, lack of effort, and/or lack of funding to support Indigenous peoples’ being involved in research events outside of their community is discouraging to Indigenous knowledge holders and engenders mistrust in research more generally. Conversely, a collaborative approach to research seeks to fully include Indigenous knowledge holders and actively acknowledge their contributions as full partners in research.

In contrast, an integrated KT approach holds the promise of more fully recognizing the partnership between researchers and knowledge users and the contributions both make in the relationship. In a research study focused on developing an intervention to support people to make what they define as good health decisions [34], the integrated KT approach involved full collaboration between the Minwaashin Lodge community and researcher partners of which JJ was the primary academic research contact. This collaboration between partners together identified the research focus and then continued throughout the entire research study to define and uphold ethical practices and to co-create knowledge.

The research process was found to accommodate both Indigenous and Western-informed perspectives in an inclusive community-research partnership that fully recognized the contributions of all parties [42]. In this study, the community-researcher partnership chose to form an advisory group consisting of Indigenous and non-Indigenous members. This advisory group established the study’s overarching goals and explored the tension between Indigenous approaches to knowledge acquisition and Western research approaches. The mutual learning within the advisory group meant that there was co-creation of evidence in ways identified by advisory group members as ethical and of relevance to Indigenous and non-Indigenous people [34]. We found that integrated KT structures collaboration between researchers and knowledge users in ways that are more likely to result in a context-sensitive research approach that is respectful and relevant, and fosters meaningful relationships based on mutual trust and respect.

### How to define and uphold ethical practices: The process is more important than the results

KT becomes an integral part of ethical research conduct when research-related relationships are experienced as a partnership that does not privilege researchers over Indigenous people or communities. Of course, end-of-project KT may be necessary to reach people who were not directly involved in a research project, but the integration of ethical processes into research that are defined by Indigenous peoples themselves as respectful and inclusive is critical. We have found that to achieve this type of research requires a focus on *how* research is conducted. In other words, research must invest in defining and then upholding ethical practices, from within a partnership with Indigenous knowledge user partners. In the process of developing this paper, authors IC and AP asked (Table 2):

- Why is research important anyway?
- Why not use the *soft moccasin* approach?
- Why don’t researchers stay around long enough to be part of some type of change from the research?

**Table 2:** Collaboration

Questions Aimed at Researchers	Recommendations
Why is research important anyway?	• People being invited to be part of research usually want to know why researchers are interested in the research, and to share in these reasons together. • People have different views on how research will improve peoples’ lives. People must make their view(s) clear and transparent from the beginning of any research process. • People need to feel safe to be able to talk and share their experiences and knowledge. To share experiences and knowledge, people need to be clear on and understand their partners’ reasons for the research, and what it is that people can accomplish together, in the research.
Why not use the *soft moccasin* approach?	• The *soft moccasin* approach is about building relationships and bridging cultural differences. This approach has no room for a person of authority, only people who want to work in partnership.
Why don’t researchers stay around long enough to be part of some type of change from the research?	• We need to discuss with researchers how implementing change can start before the project timeline is finished. • Researchers need to ask how they can be actively involved in sharing with the community. While academics get recognized for acquiring research grants and publishing papers, they need to consider how they can give back to the community and what they can meaningfully offer to community partners. • Researchers need to incorporate and consider how end-of-project findings are communicated in ways that directly benefit the community.

Researchers must conduct themselves in ways that demonstrate trust, integrity, and an interest in developing an equitable relationship with Indigenous groups and communities. Such an effort requires more than a commitment to follow research principles such as the Ownership, Control, Access and Possession (OCAP®) [21] of research, that govern and protect the rights of self-determination by Indigenous people within research. It also involves an investment of time in the community to learn and understand how research principles will be applied on the ground in practical terms.

For example, in the FASD study that involved MMN and AP, the findings of the study were shared in multiple forms with the purpose of demonstrating the community/researcher partnership: the findings were put into a community report that was shared with people involved in FASD work; presentations were made in the community with participants and program managers who could enact recommendations; and, the community report was included in the appendix of MMN’s doctoral dissertation. While some of the findings and recommendations from the FASD study were incorporated into community-led action plans, it was MMN’s involvement in developing some recommendations that garnered more attention and support in the community, leading to more projects and stronger relationships in the subsequent years.

### Indigenous KT and integrated KT as complementary KT approaches: Expanding ways of knowing

KT that is ‘by and with’ Indigenous peoples is referred to as “Indigenous KT” [13]. It has been defined and described in different ways, though in every instance it emphasizes sharing knowledge in ways that are locally developed and contextualized. Smylie et al. [43] identify that Indigenous KT is about “sharing what we know about living a good life” (p. 16) and assert that research must find ways to meaningfully include Indigenous ways of knowing and doing. Such ways of knowing and doing can be articulated through the explicit recognition of Indigenous knowledge systems and affirmation of principles that are specific to Indigenous groups [21, 44, 45]. Nevertheless, while KT activities are widely considered a requirement in contemporary research designs, there remains a dearth of published literature on how to practice Indigenous KT [46]. We assert that Indigenous KT is an approach to knowledge production that is defined and led by Indigenous people involved in research, and it is therefore underpinned by local Indigenous worldviews. While it is a distinct approach to the collaborative creation and sharing of relevant and useful knowledge by and with Indigenous people, it can be said to share some similarities with a Western-informed approach of integrated KT. In many ways and while remaining distinct, the ideals and objectives of an integrated KT approach in research appear congruent with and complimentary to those of Indigenous KT despite the distinct world views and values of each approach to KT. For example, both are done ‘by and with’ knowledge users, and both emphasize consideration of contextual information in the creation of useful, relevant and applicable knowledge. Indigenous KT is the domain of an Indigenous community and its use is led by holders of Indigenous knowledge. Integrated KT is therefore, with the approval of Indigenous community partners, an approach that is appropriate for use by researchers who hold Western knowledge and work in academic and health settings, and who also seek to ethically and effectively engage with Indigenous community partners.

## Conclusion

An integrated KT approach is rooted in and committed to prioritizing researcher-community relationships and creating an ethical space for different forms of knowledge. These qualities are imperative to *all* Indigenous health research, as they ensure that both key guidelines for ethical research with Indigenous peoples and Indigenous principles of research are integrated into all facets of the research activity. There are assumptions that institutional academic settings such as universities are places for different knowledge systems to coexist, propagate and benefit from introspection. We assert that integrated KT is a valuable research approach that can facilitate opportunities for different forms of knowledge to be generated and flourish within our society and to its benefit.

## Abbreviation

KT: Knowledge Translation

## Footnotes

1 OCAP® is a registered trademark of First Nations Information Governance Centre and is further defined at www. FNIGC.ca/OCAP.
